# No correlation between femoral tunnel orientation and clinical outcome at long-term follow-up after non-anatomic anterior cruciate ligament reconstruction

**DOI:** 10.1007/s00167-019-05366-w

**Published:** 2019-02-01

**Authors:** David Sundemo, Julia Mårtensson, Eric Hamrin Senorski, Eleonor Svantesson, Jüri Kartus, Ninni Sernert, Jón Karlsson, Kristian Samuelsson

**Affiliations:** 1 Department of Orthopaedics, Institute of Clinical Sciences, The Sahlgrenska Academy, University of Gothenburg, Gothenburg, Sweden; 2 NU-Hospital Group, Trollhättan/Uddevalla, Sweden; 3 Department of Orthopaedics, Sahlgrenska University Hospital, Mölndal, Sweden

**Keywords:** Anterior cruciate ligament reconstruction, Osteoarthritis, Radiographs, Surgery, Outcome, Femoral tunnel angle, Quadrant method

## Abstract

**Purpose:**

This study aimed to determine the influence of femoral tunnel orientation on long-term clinical outcome and osteoarthritis in patients undergoing ACL reconstruction and to test the reliability of the implemented radiographic measurement methods. It was hypothesized that a more horizontal femoral tunnel would correlate with superior clinical outcome.

**Methods:**

A cohort of 193 patients who underwent non-anatomic ACL reconstruction was examined. In this specific study, non-anatomic is defined by the surgeons’ pursuit of optimal isometry, not to emulate the native ACL anatomy. At follow-up, the Lachman test, the KT-1000, the pivot-shift test, the one-leg-hop test and the IKDC-2000 were evaluated. Osteoarthritis was evaluated radiographically. Posteroanterior and lateral radiographs were used to determine the position of the femoral tunnel in the coronal and sagittal planes and the angle of the tunnel in the coronal plane. A method for determining femoral rotation on the lateral radiographs was developed and its reliability was evaluated. The femoral tunnel orientation was analyzed to examine its influence on clinical outcome and osteoarthritis.

**Results:**

A total of 101 patients were analyzed at a mean of 16.4 (± 1.3) years postoperatively. The reliability of the measurement methods was regarded as good to excellent (ICC 0.57–0.97). The mean coronal femoral tunnel angle was 9.6° (± 9.4°). The coronal femoral tunnel was positioned at a mean of 43% (± 3.5%) of the distance measured from lateral to medial. The mean sagittal femoral tunnel position, measured using the quadrant method, was 40% (± 6.4%) from posterior to anterior. No significant associations were found between tunnel orientation and the clinical outcome variables.

**Conclusions:**

The orientation of the femoral tunnel did not predict the long-term subjective outcome, functional outcome or the development of osteoarthritis in patients undergoing non-anatomic ACL reconstruction. The method for determining femoral rotation on lateral radiographs was found to be reliable.

**Level of evidence:**

Retrospective cohort study, level of evidence IV.

## Introduction

The position and angle of the bone tunnels in anterior cruciate ligament (ACL) reconstruction are central aspects, which can affect laxity restoration, loss of motion and the future risk of developing osteoarthritis (OA) [11, 26]. Incorrect tunnel placement has been shown to be a common cause of technical failure in ACL reconstruction [23, 25, 32, 33].

During the past decade, there has been a shift from isometric, non-anatomic ACL reconstruction, often performed using the transtibial (TT) technique, to a more anatomic approach, which could be performed using transportal (TP) techniques [9]. The graft in anatomic ACL reconstruction is oblique and horizontal, in both the coronal and sagittal planes [14]. As a result, it has been suggested that anatomic ACL reconstruction improves the rotational laxity of the knee and consequently decreases the pivot-shift phenomenon [1, 20]. In anatomic ACL reconstruction, the femoral tunnel is placed using either a single- or double-bundle technique, aiming at the native femoral footprint with the aid of intra-articular anatomic landmarks [1]. As a result, the differences between non-anatomic and anatomic ACL reconstruction are predominantly the position and angle of the bone tunnels. To investigate the influence of tunnel orientation, several previous studies have examined postoperative tunnel placement radiographically with regard to clinical outcome [11, 26, 29]. A more horizontal femoral tunnel in the coronal plane has been associated with superior results in terms of rotatory knee laxity, the development of OA and subjective outcome [16, 26]. However, to the authors’ knowledge, no study to date, with more than 10 years of follow-up, has assessed the long-term influence of femoral tunnel orientation on the clinical outcome.

A review of the literature reveals that various methods for the postoperative radiographic assessment after ACL reconstruction have been developed and utilized, but only a few have been tested with regard to reliability [2, 12, 14, 26]. The reliability of radiographic techniques in determining tunnel orientation varies between studies, making it important to validate measurement reliability in this field. One issue complicating the assessment of radiographs is to determine the amount of rotation of the distal femur seen on standard lateral radiographs. A sufficient overlap of the femoral condyles is desirable to ensure a lateral projection. Arbitrary methods have frequently been used to determine whether an adequate projection has been obtained. The use of fluoroscopy, where true lateral projections can be assured, is a good alternative, although this technique is not always available.

The purpose of this study was to determine the influence of femoral tunnel orientation on long-term clinical outcome and osteoarthritis in patients undergoing ACL reconstruction and to test the reliability of the implemented radiographic measurement methods. Moreover, the purpose was to test the reliability of a new method that was created to standardize the analysis of femoral rotation on lateral radiographs. The hypothesis of the study was that patients with more horizontal femoral tunnels, in both the coronal and sagittal projections, would experience a superior clinical outcome and less OA in the long term.

## Materials and methods

In this retrospective analysis of two previously published randomized, controlled trials [7, 19], a total of 193 patients were recruited from three medical centers in Sweden. Patients with an ACL rupture and only minor chondral lesions (Outerbridge grades 1 to 2) were included. Meniscal injuries were not considered a reason for exclusion. Patients suffering from major chondral lesions, multiligament injuries or a previous ACL reconstruction of the ipsilateral or contralateral knee were excluded from the study. ACL reconstructions were performed by one of six experienced senior surgeons and patients were divided into three groups receiving an ipsilateral patellar tendon autograft, an ipsilateral three-strand semitendinosus autograft or an ipsilateral four-strand semitendinosus/gracilis autograft. Reconstructions were performed from September 1995 through January 2000. The surgical technique and postoperative rehabilitation protocol have been described in detail in a previous study [4]. The term “non-anatomic ACL reconstruction” is used to explain that the surgeons did not pursue native ACL anatomy but instead aimed to create graft isometry, which was a common method at the time the study was initiated. The study was approved by the Human Ethics Committee at Gothenburg University and Stockholm University. Written informed consent was obtained from all patients.

### Clinical assessments and follow-up

Preoperative and postoperative assessments were performed by a research assistant or physical therapists who were not involved in the surgery or rehabilitation. Anteroposterior knee laxity was measured using the Lachman test (graded as 0, 1+, 2+ or 3+) and using a KT-1000 arthrometer [6] (MEDmetric San Diego, CA) to perform the manual maximum test. To assess rotatory knee laxity, the standardized pivot-shift test [10], developed from the original technique presented by Galway and MacIntosh, was used [8]. The pivot-shift test was graded according to IKDC criteria as normal, glide, clunk and gross (or 0, 1+, 2+, 3+). Both the Lachman test and the pivot-shift test were dichotomized for the purpose of analysis. Dichotomization was performed to differentiate between patients with totally stable knees and patients with instability, independent of the degree of instability. Consequently, the two subgroups contain patients with grade 0 vs. patients with grade 1+, 2+ and 3+. To evaluate knee function, the one-leg-hop test [31] was used and compared with the contralateral side using a quotient. Subjective outcome was assessed using the IKDC 2000 subjective knee evaluation form [15].

### Radiologic assessment

At the long-term follow-up, bilateral weight-bearing radiographs with the knee in 30–45° of flexion were obtained in the posteroanterior, lateral and patellofemoral projections. To grade osteoarthritis of the knees, the Kellgren–Lawrence classification was used [17]. Grading was performed by one senior radiologist with extensive experience of orthopedic radiographic evaluations. For the purpose of analysis, patients were dichotomized into subgroups with either none to mild (0–1) or moderate to severe (2–4) radiographic signs of osteoarthritis. To validate interrater reliability, the positions and angles of the tunnels were reviewed independently by two of the authors. Further, 2 weeks after the first review, the measurements were repeated to determine intraobserver reliability. Measurements were made using Centricity Enterprise Web V4.0 (General Electric Healthcare, Barrington, Illinois, USA), a software program that enables the determination of angles and distances. In the final analysis, only measurements from the first review by one examiner were used.

**Fig. 1:**
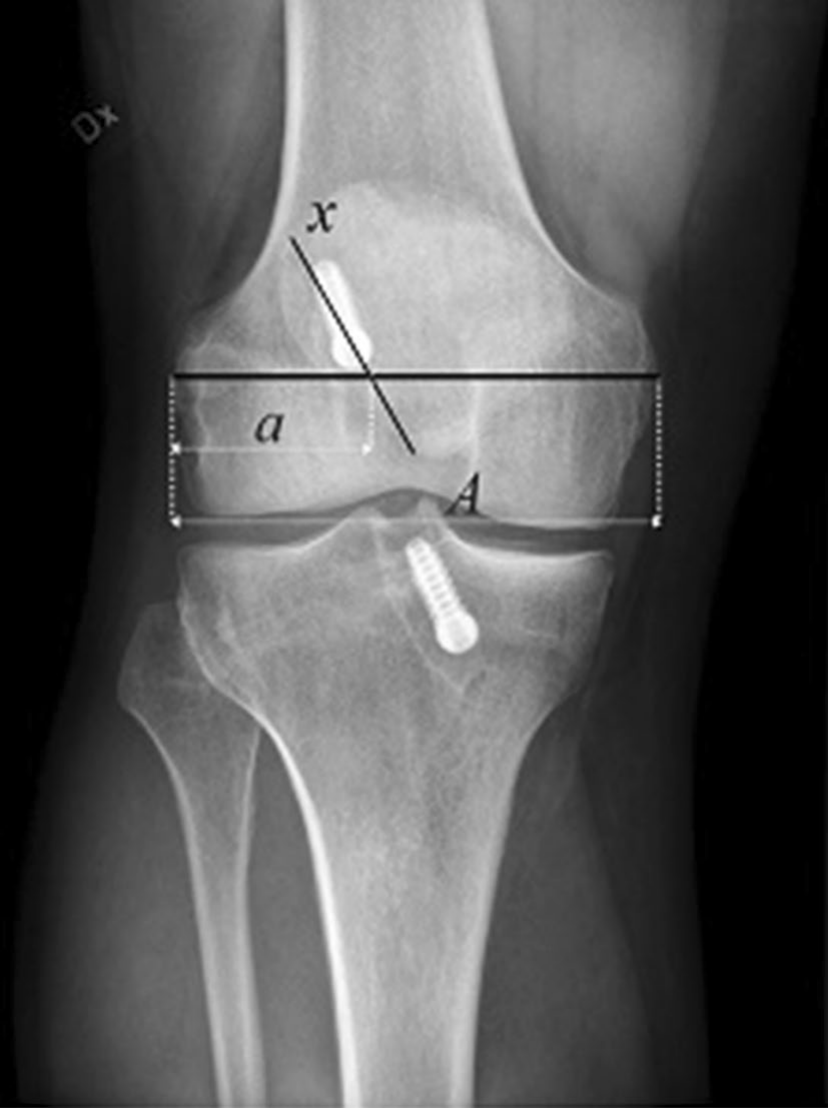
A radiograph showing the measurement technique for the femoral tunnel position in the coronal plane. The largest distance between the medial and lateral femoral condyles was identified (A). The distance from the lateral condyle to the center of the tunnel was measured (a). The obtained distance (a) was divided by the total width (A) and the position of the femoral tunnel was expressed as a percentage

The position of the femoral tunnel in the coronal plane was measured on the posteroanterior radiograph. The largest distance between the medial and lateral femoral condyles was identified (A). The distance from the lateral condyle to the center of the tunnel was measured (a). The obtained distance (a) was divided by the total width (A) and the position of the femoral tunnel was expressed as a percentage (Fig. 1).

**Fig. 2:**
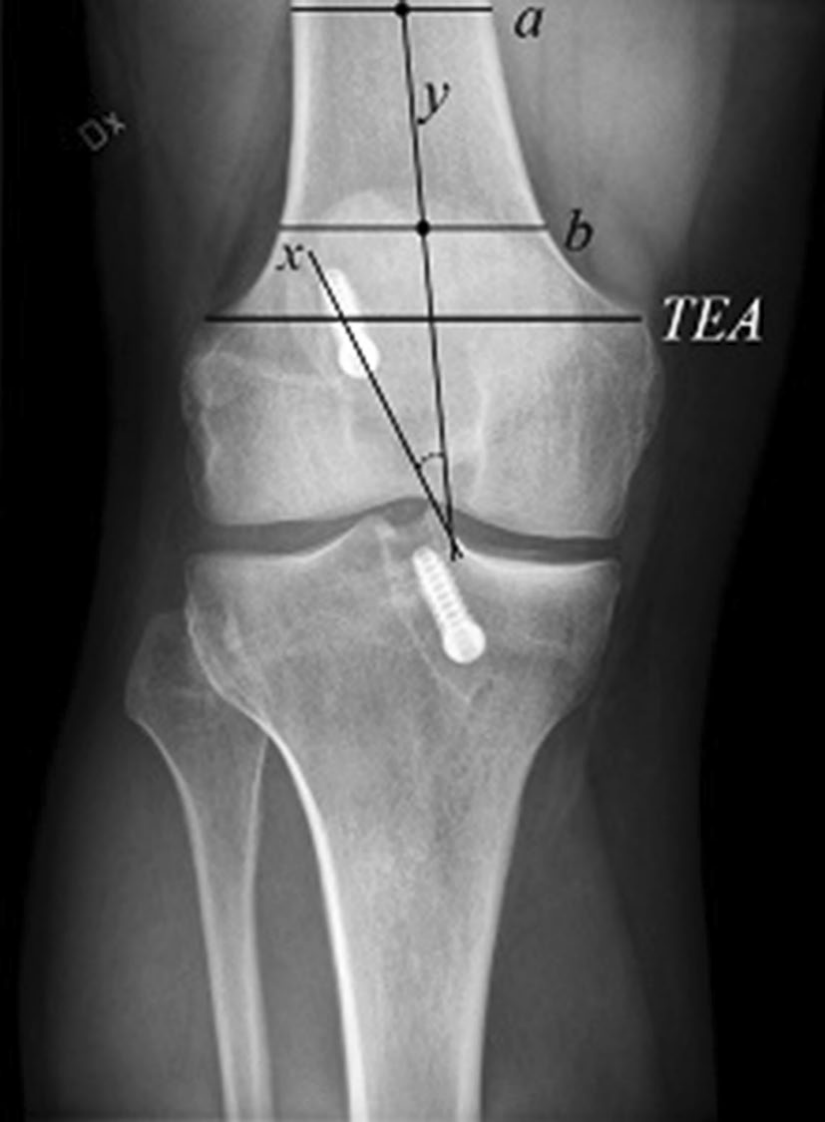
A radiograph showing the method used to measure the femoral tunnel angle formed by the femoral tunnel and the long axis of the femur in posteroanterior radiographs. The most proximal portion of the femoral epicondyle was bisected by a line forming the transepicondylar axis (TEA). At the most proximal part of the femur, visualized on the radiograph, a line was drawn along the width of the diaphysis and parallel to the TEA (a). Distal to this line, at a distance from line a that corresponds to half the length of the TEA, another parallel line was drawn (b). The midpoint of lines a and b was crossed by a line parallel to the axis of the diaphysis. The femoral tunnel was visualized and a line was drawn through its axis (*x*). The angle formed between this line and line y was defined as the femoral tunnel angle

To evaluate the angle of the femoral tunnel in the coronal plane, a method recently published by Illingworth et al. [14] was implemented. The most proximal portion of the femoral epicondyle was bisected by a line forming the transepicondylar axis (TEA). At the most proximal part of the femur, visualized on the radiograph, a line was drawn along the width of the diaphysis and parallel to the TEA (a). Distal to this line at a distance of half the length of the TEA, another parallel line was drawn (b). The midpoint of lines *a* and *b* was crossed by a line parallel to the axis of the diaphysis. The femoral tunnel was visualized and a line was drawn through its axis (*x*) (Fig. 2). The angle formed between this line and line y was defined as the femoral tunnel angle. In a few patients, only a short part of the femur was visible. In these patients, the b-line on occasion was distal to the TEA, in which case the analysis for that particular patient was excluded.

**Fig. 3:**
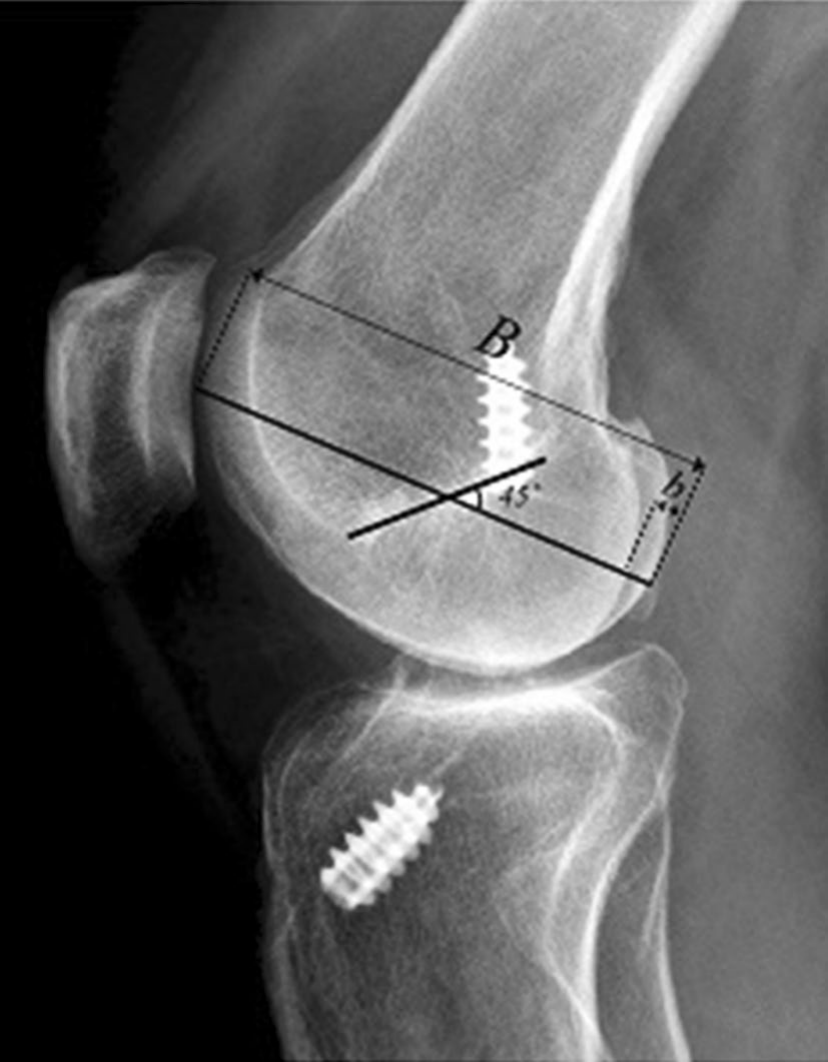
The radiograph shows the newly developed femoral condyle overlap method, used to validate rotation for lateral radiographs. A line was drawn from the posterior to the anterior edge of the femoral condyle (B), intersecting the midpoint at a 45° angle. The angle size was determined as 45°, since it was estimated to represent the anteroposterior axis of the femoral condyle. Moreover, a line (b) was drawn from the posterior limit of the femoral condyle to the border of the femoral condyle situated just anteriorly (corresponding to the medial or lateral condyle depending on the direction of the rotation of the knee), whereafter the femoral condyle overlap can be calculated using an equation

**Fig. 4:**
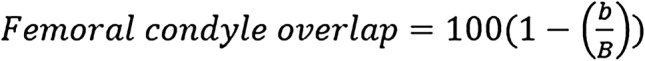
Formula used to calculate the femoral condyle overlap

Lateral radiographs were utilized to measure the position of the femoral tunnel in the sagittal plane. When analyzing lateral radiographs, it is important to note and, if necessary, exclude radiographs not obtained from a straight lateral direction. This is ensured by confirming sufficient overlap of the femoral condyles. During the planning of the present study, a method was developed to assess femoral condyle overlap and exclude radiographs not meeting the predetermined criteria (Fig. 3). The first step in the method was to find the center of Blumensaat’s line, which was established by measurement. A line was drawn from the posterior to the anterior edge of the femoral condyle (B), intersecting the midpoint at a 45° angle. The angle size was determined as 45°, since it was estimated to represent the anteroposterior axis of the femoral condyle. Moreover, a line (b) was drawn from the posterior limit of the femoral condyle to the border of the femoral condyle situated just anteriorly (corresponding to the medial or lateral condyle depending on the direction of the rotation of the knee). The femoral condyle overlap was calculated using the formula shown in Fig. 4.

Radiographs were included in the final analysis, assessing the influence on the clinical outcome variables if the overlap of the condyles was larger than 90%, a degree of overlap accepted by Sullivan et al. [30] in a previous study.

**Fig. 5:**
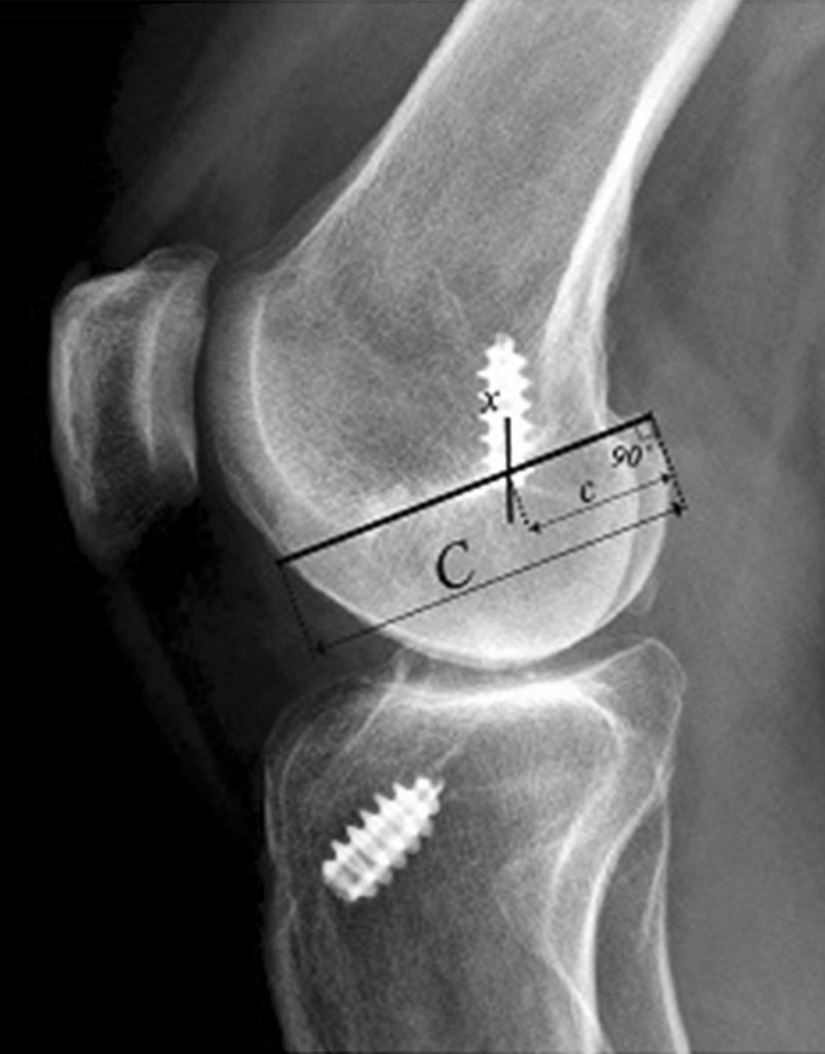
A radiograph showing the method used to measure the position of the femoral tunnel according to the quadrant method. A grid is placed over the lateral femoral condyle and the superior border is aligned with the Blumensaat line and the inferior border with the distal end of the femoral condyle. The borders of the grid in the anteroposterior directions consist of the most anterior and posterior ends of the femoral condyle, forming distance C. Distance c is divided by distance C and the position is presented as a percentage from posterior to anterior (Fig. 4)

An evaluation of the femoral tunnel in the sagittal plane was made using a modified version of the quadrant method [2]. The quadrant method, developed by Bernard and Hertel [2], is widely used in assessments of native and reconstructive ACL anatomy [2, 3, 22, 24]. In the original version, a grid is placed over the lateral femoral condyle, with the superior border aligned with Blumensaat’s line and the inferior border with the distal end of the femoral condyle. The borders of the grid in the anteroposterior direction consist of the most anterior and posterior ends of the femoral condyle. However, in the present study, only the anteroposterior position could be evaluated, since there were no radiopaque markers depicting the aperture of the tunnel in the supero-inferior direction. In the present study, the position of the femoral tunnel in the sagittal plane, along Blumensaat’s line, is presented as a percentage from posterior to anterior (Fig. 5).

### Statistical analysis

Analyses of intrarater and interrater reliability were made using intraclass correlation coefficients (ICC) [28]. Classifications of reliability were made in accordance with Shoukri and Pause [27] as poor (< 0.4), good (0.4–0.75) and excellent (> 0.75). To analyze dependent variables as a result of the measured radiographic angles and positions, multiple linear regression analysis was used. The dependent variables were assumed to be linearly correlated to the independent variables. Analysis with regard to surgical portal access was performed with Fisher’s exact test, the Chi square exact test and the Mann–Whitney *U* test for dichotomous, ordered categorical and continuous variables, respectively. Value intervals are presented with means and standard deviations, to illustrate differences in outcome depending on graft placement. The selection of the particular intervals was made to divide the patients into three equally sized groups. The continuous and dichotomized outcome variables are presented using statistical beta and odds ratios, respectively. The estimate of the statistical analysis is presented using 95% confidence intervals for both continuous and dichotomized variables.

## Results

**Fig. 6:**
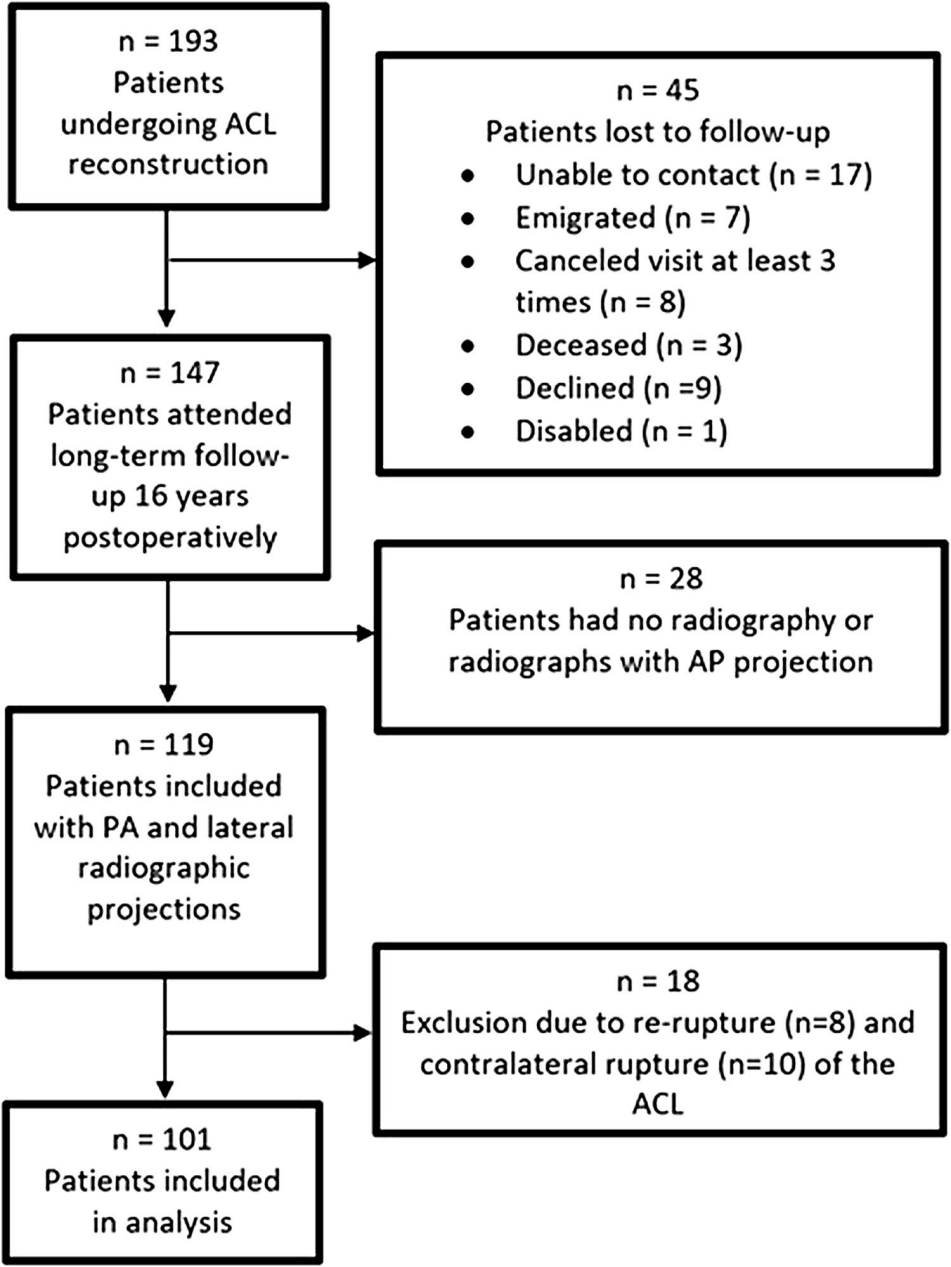
Flow chart of included patients. *ACL* anterior cruciate ligament, *PA* posteroanterior, *AP* anteroposterior

Of the 193 patients enrolled in the two original randomized, controlled trials, 147 (76%) attended the long-term follow-up at a mean of 16 years postoperatively [4]. Twenty-eight (12%) patients did not undergo a complete radiographic assessment at follow-up. To standardize the analysis, these patients were excluded. Eight and ten patients sustained a re-rupture and a contralateral rupture of the ACL, respectively. Pre-revision radiographs were available for six of the patients who sustained a re-rupture, although these radiographs were not included in the final analysis. Consequently, the radiographs of 101 (52%) patients were assessed individually for each parameter needed for inclusion in the study (Fig. 6). Using the femoral condyle overlap method, 18 (9%) patients had to be excluded from the quadrant method analysis. Moreover, when assessing the femoral tunnel angle, the b-line was found to be distal to the TEA in 10 (5%) patients, leading to their exclusion from analysis. The use of autograft type was similar in the analyzed cohort. The transtibial portal technique was considerably more frequent (*n* = 72, 71%) compared with the transportal technique [*n* = 29, 29% (Table 1)].

**Table 1:** Demographics of patients at long-term follow-up

Age at operation, years	28.7 (8.7), *n* = 101
Gender, male/female	69/32, *n* = 101
Follow-up period, years	16.4 (1.3), *n* = 101
Surgical portal technique	
Transportal technique	29 (29%)
Transtibial technique	72 (71%)
Type of graft	
Bone-patellar tendon-bone autograft	46 (45.5%)
Hamstring tendon autograft	55 (55.5%)
Associated injuries, dichotomized	
No	30 (30%)
Yes	71 (70%)
Femoral angle, coronal, degrees	9.6° (9.4°), *n* = 91
Quadrant method, %	40 (6.4), *n* = 83
Femoral tunnel position, coronal, %	43 (3.5), *n* = 100
One-leg-hop test, limb symmetry index	91 (19.3), *n* = 92
IKDC 2000 form^a^, total score	71 (20.7), *n* = 101
KT 1000 MMT^b^, mm, side-to-side difference	1.7 (3.0), *n* = 101
Kellgren–Lawrence, injured side	
0	29 (29%)
1	29 (29%)
2	28 (27%)
3	10 (10%)
4	5 (5%)
Pivot-shift test, dichotomized	
Pivot shift: 0	64 (64%)
Pivot shift: 1,2,3	36 (36%)
Lachman test, dichotomized	
Lachman 0	49 (48.5%)
Lachman 1,2,3	52 (51.5%)

For categorical variables, *n* (%) is presented. For continuous variables, the mean (SD) is presented

^a^International Knee Documentation Committee subjective knee evaluation form

^b^KT-1000 arthrometer manual maximum test

Intra- and interrater reliability were considered excellent for the femoral tunnel angle in the coronal plane (ICC: 0.83 and 0.94), lateral condyle overlap (ICC: 0.97 and 0.90) and for the quadrant method (ICC: 0.88 and 0.80). When analyzing the reliability of the femoral tunnel position in the coronal plane, the intrarater and interrater reliability were considered good (ICC 0.72 and 0.57, respectively, Table 2).

**Table 2:** Intraclass correlation coefficients for intrarater and interrater reliability

Measurement	Intrarater reliability ICC (95% CI)	Interrater reliability ICC (95% CI)
Femoral tunnel angle	0.83 (0.75–0.88)	0.94 (0.87–0.97)
Femoral tunnel position, coronal	0.72 (0.61–0.80)	0.57 (0.26–0.77)
Femoral condyle overlap	0.97 (0.95–0.98)	0.90 (0.79–0.95)
Quadrant method	0.88 (0.82–0.92)	0.80 (0.44–0.92)

*ICC* Intraclass correlation coefficient, *CI* Confidence interval

Multiple linear regression analysis revealed that neither the angle of the femoral tunnel (Table 3), the position of the femoral tunnel in the coronal plane (Table 4) nor the position of the femoral tunnel in the sagittal plane, assessed using the quadrant method (Table 5), was significantly associated with the assessed outcome variables.

**Table 3:** Adjusted multiple linear regression analysis for the femoral tunnel angle

		One-leg-hop test, mean (SD)	IKDC 2000, total score (SD)	KT-1000 MMT side-to-side difference (SD)	Pivot shift dichotomized (0 vs. 1+, 2+, 3+), *n* (%) of events	Lachman test dichotomized (0 vs. 1+, 2+, 3+), *n* (%) of events	Kellgren–Lawrence dichotomized (0–1 vs. 2–4), *n* (%) of events
FTA, value intervals	− 28–< 6	88 (15.6)	69 (19.1)	2.2 (3.0)	9 (30%)	18 (58%)	15 (48%)
	6–< 12.6	94 (18.6)	77 (15.9)	2.2 (2.9)	7 (23%)	14 (47%)	13 (43%)
	12.6–38.8	93 (22.3)	71 (24.8)	1.1 (3.1)	15 (50%)	15 (50%)	11 (37%)
FTA, beta (95% CI)		− 0.23 (− 0.71;0.24)	− 0.35 (− 0.81;0.12)	− 0.04 (− 0.11;0.03)	Not applicable	Not applicable	Not applicable
FTA, OR (95% CI)		Not applicable	Not applicable	Not applicable	1.05 (0.99–1.11)	1.0 (0.95–1.05)	1.01 (0.96–1.07)
FTA, *p*-value		n.s	n.s	n.s	n.s	n.s	n.s
Missing patients^a^		10	10	10	10	10	10

Multivariate analysis adjusted for the type of graft, surgical portal, associated injuries and age at operation. For the dichotomized variables, the number of events and the percentage of the total number of patients in that particular value interval are presented

*SD* Standard deviation, *IKDC* International Knee Documentation Committee, *MMT* manual maximum test, *FTA* femoral tunnel angle, *CI* confidence interval, *OR* odds ratio, *Ns* not significant

^a^Missing patients are patients with incomplete data for the particular analysis, details regarding this can be found in the results section

**Table 4:** Adjusted linear regression analysis for the femoral tunnel position in the coronal plane

		One-leg-hop test, mean (SD)	IKDC 2000, total score (SD)	KT-1000 MMT side-to-side difference (SD)	Pivot shift dichotomized (0 vs. 1+, 2+, 3+), *n* (%) of events	Lachman test dichotomized (0 vs. 1+, 2+, 3+), *n* (%) of events	Kellgren–Lawrence dichotomized (0–1 vs. 2–4), n (%) of events
FTP coronal, value intervals	31.2–< 41.5	95 (26.5)	72 (20.3)	1.9 (2.8)	9 (27%)	16 (49%)	11 (33%)
	41.5 < 44.9	93 (15.0)	74 (19.1)	1.9 (2.6)	15 (46%)	18 (53%)	15 (44%)
	44.9–48.9	87 (14.4)	69 (23.1)	1.5 (3.5)	11 (33%)	18 (55%)	17 (52%)
FTP coronal, beta (95% CI)		− 0.88 (− 2.10;0.34)	0.70 (− 0.43;1.84)	− 0.01 (− 0.18;0.17)	Not applicable	Not applicable	Not applicable
FTP coronal, OR (95% CI)		Not applicable	Not applicable	Not applicable	1.04 (0.92–1.18)	1.01 (0.90–1.14)	0.95 (0.83–1.08)
FTP coronal, *p*-value		N.s	N.s	N.s	N.s	N.s	N.s
Missing patients^a^		1	1	1	1	1	1

Multivariate analysis adjusted for the type of graft, surgical portal, associated injuries and age at operation. For the dichotomized variables, the number of events and the percentage of the total number of patients in that particular value interval are presented

*SD* Standard deviation, *IKDC* International Knee Documentation Committee, *MMT* manual maximum test, *FTA* femoral tunnel angle, *CI* confidence interval, *OR* odds ratio, *Ns* not significant

^a^Missing patients are patients with incomplete data for the particular analysis, details regarding this can be found in the results section

**Table 5:** Adjusted linear regression analysis for the quadrant method

		One-leg-hop test, mean (SD)	IKDC 2000, total score (SD)	KT-1000 MMT side-to-side difference (SD)	Pivot shift dichotomized (0 vs. 1+, 2+, 3+), *n* (%) of events	Lachman test dichotomized (0 vs. 1+, 2+, 3+), *n* (%) of events	Kellgren–Lawrence dichotomized (0–1 vs. 2–4), *n* (%) of events
Quadrant method, value intervals	27.3–< 37.5	93 (18.7)	78 (14.0)	2.0 (2.7)	11 (39%)	14 (52%)	8 (30%)
	37.5–< 42.9	91 (13.2)	67 (23.4)	1.6 (3.3)	11 (41%)	12 (43%)	11 (39%)
	42.9–57.8	97 (22.8)	74 (20.0)	1.5 (3.1)	10 (37%)	17 (61%)	13 (46%)
Quadrant method, beta (95% CI)		0.41 (− 0.28;1.11)	− 0.03 (− 0.69;0.63)	0.01 (− 0.10;0.12)			
Quadrant method, OR (95% CI)					0.95 (0.88–1.03)	1.00 (0.93–1.08)	1.04 (0.96–1.12)
Quadrant method, *p*-value		n.s	n.s	n.s	n.s	n.s	n.s
Missing patients^a^		18	18	18	18	18	18

Multivariate analysis adjusted for the type of graft, surgical portal, associated injuries and age at operation. For the dichotomized variables, the number of events and the percentage of the total number of patients in that particular value interval are presented

*SD* Standard deviation, *IKDC* International Knee Documentation Committee, *MMT* manual maximum test, *FTA* femoral tunnel angle, *CI* confidence interval, *OR* odds ratio, *Ns* not significant

^a^Missing patients are patients with incomplete data for the particular analysis, details regarding this can be found in the results section

All patients with a bone-patellar tendon-bone autograft were reconstructed using the transtibial technique. Patients with a hamstring tendon autograft underwent reconstruction using either the transtibial (26 patients) or the transportal (29 patients) techniques. Patients undergoing ACL reconstruction using the transtibial technique were examined later (mean 16.9 ± 0.8 years) than patients undergoing reconstruction using the transportal technique (mean 15.1 ± 1.3 years), *p*  ≤ 0.0001.

Using the quadrant method, patients reconstructed using a transportal technique had tunnels that were located more posteriorly along Blumensaat’s line (mean 38 ± 5.6%) than patients in whom the transtibial technique was utilized (mean 41 ± 6.5%). However, the result was not statistically significant. Similarly, the surgical portal that was used had no significant influence on the femoral tunnel angle (TP: 11.0° ± 8.6, TT: 8.9° ± 9.7, Table 6).

**Table 6:** Comparisons by surgical portal

	Transportal technique (*n* = 29)	Transtibial technique (*n* = 72)	*p* value
Type of graft			
Bone-patellar tendon-bone autograft	0 (0%)	46 (64%)	< 0.0001
Hamstring tendon autograft	29 (100%)	26 (36%)	
Age at operation, years	26.3 (6.2)	29.7 (9.4)	n.s
Follow-up period, years	15.1 (1.3)	16.9 (0.8)	< 0.0001
Associated injuries, dichotomized			
No	8 (28%)	22 (31%)	n.s
Yes	21 (72%)	50 (69%)	
Femoral angle, coronal, degrees	11.0 (8.6)	8.9 (9.7)	n.s
Quadrant method, %	38 (5.6)	41 (6.5)	n.s
Femoral tunnel position, coronal, %	43 (3.5)	43 (3.6)	n.s
Kellgren–Lawrence, injured side			
0	8 (28%)	21 (29%)	n.s
1	9 (31%)	20 (28%)	
2	9 (31%)	19 (26%)	
3	3 (10%)	7 (10%)	
4	0 (0%)	5 (7%)	
Pivot-shift test, dichotomized			
Pivot shift: 0	22 (76%)	42 (69%)	n.s
Pivot shift: 1,2,3	7 (24%)	29 (31%)	
Lachman test, dichotomized			
Lachman 0	15 (52%)	34 (47%)	n.s
Lachman 1,2,3	14 (48%)	38 (53%)	

Analysis with regard to surgical portal technique. For categorical variables, *n* (%) is presented. For continuous variables, the mean (SD) is presented

*Ns* not significant

## Discussion

The most important finding was that the placement and angle of the femoral tunnel after non-anatomic ACL reconstruction did not predict the clinical, subjective or radiographic outcome at a mean of 16 years postoperatively, but the measurement methods were found to be reliable. Contrary to our results, Pinczewski et al. [26] performed a study of 200 patients who underwent clinical and radiologic assessment 7 years after ACL reconstruction. The authors reported that a more vertical graft in the coronal plane was significantly correlated to inferior rotational stability and an increased level of OA. A similar but non-identical measurement methodology was used in their study, which could partly explain the differences. Moreover, Pinczewski et al. [26] only included patients with hamstring tendon grafts. One important difference is that the mean follow-up period in the present study was 16 years compared with 7 years in the study by Pinczewski et al. [26]. It is well known that ACL injury is associated with an increased risk of developing OA [21]. In time, differences in function and stability could possibly level out, as a result of the general joint degeneration.

The present study found no significant differences in outcome when patient groups were compared based on the surgical portal technique. However, patients who underwent transtibial ACL reconstruction had tunnel apertures that were more anteriorly placed along the Blumensaat line compared with those who underwent the transportal technique. This was probably a result of the restriction caused by the angle of the tibial tunnel when the drilling of the femoral tunnel was performed. A recent systematic review established that the mean position of the native femoral insertion was 28.4% ± 5.1% from the posterior border when using the quadrant method [34]. Consequently, in the present study, the transportal technique displayed a tendency towards producing femoral tunnels closer to the native ACL anatomy. Previous studies have also shown that the femoral tunnel angle is more horizontal in patients reconstructed using transportal techniques [5, 14]. In the present study, the femoral tunnel angle was not affected by the choice of surgical portal. The probable reason for this is that the patients were reconstructed prior to the implementation of the transportal anatomic ACL reconstruction and the surgeons strove to achieve optimal isometry not anatomy. The opportunity to utilize the increased maneuverability inherent to the transportal approach was therefore not taken.

The femoral tunnel angle and the position of the femoral tunnel in the frontal and the sagittal plane were assumed to be linearly correlated to the outcome variables. This assumption was based on studies that have determined the native anatomic positions of the ACL insertion to the femur. Measurements in the current study showed that no femoral tunnel angle was larger than what was regarded as “within anatomic range” by the originators of the utilized method [14]. Similarly, no tunnel was closer to the posterior border of the quadrant than the synthesized position presented in a recent systematic review assessing the native femoral insertion of the ACL [34]. Since anatomic ACL reconstruction is considered superior in terms of mitigating rotational laxity in the short- to mid-term perspective, the authors of the present study consider it natural to assume a linear correlation towards more anatomic positioning [13]. However, it is important to recognize that the patients in the present study, in spite of having femoral tunnels within the limits of what the reference literature considers to be within the anatomic range, were not necessarily anatomically reconstructed. Since all measurements were made in only one projection, such conclusions cannot be drawn. Moreover, to determine anatomic graft placement, both tibial and femoral tunnel positioning must be assessed.

The inter- and intrarater reliability, analyzed using intraclass correlation coefficients, of the performed measurements were considered excellent or good. These results are in line with previous studies showing that measurements of the femoral tunnel angle, [14] the quadrant method [18, 30] and the coronal femoral tunnel position [26] are reliable. Moreover, the good validity of the Bernard quadrant method has previously been established in a study comparing 3D-CT images with standard radiographs [18].

In the present study, the femoral condyle overlap method was shown to be highly reliable. Although not yet validated against other quantitative measurement methods, a similar strategy using 90% condyle-to-condyle overlap has previously been used [30]. Further, since the femoral condyle overlap method is used to specify which radiographs can be used to assess the position of the femoral tunnel in the sagittal plane, potential varus or valgus deviation would not affect the interpretation of the outcome. It, therefore, appears to be superior to evaluate knee rotation by measuring the anteroposterior axis of the femoral condyle and not the total area of the femoral condyle overlap. It is evident that the availability of an objective method to determine the rotation of lateral radiographs is useful. The method is able to determine which radiographs are of sufficient quality for research purposes. Using the fluoroscopic technique to obtain optimal lateral radiographs is superior, but, using the femoral condyle overlap analysis method, data from previous studies can be analyzed without needing to re-examine patients using fluoroscopy.

There are some limitations that need to be mentioned. First, of 193 patients, 147 (76%) attended the clinical assessment after a mean of 16 years. Regrettably, several patients had no radiographs or had incorrect projections to enable a standardized analysis. Additional patients were excluded due to re-rupture or contralateral ACL rupture. Patients with a re-rupture were excluded, since the performance of the original tunnel of the primary ACL reconstruction could no longer be assessed. Too few pre-revision radiographs were available to perform a statistically adequate separate analysis of predictors of re-rupture. Patients with a contralateral ACL injury were excluded, since the comparison between the index and the contralateral knee would be confounded. Importantly, a few possible confounders, such as age, graft type, associated injuries and the surgical portal technique, could be adjusted in order to use the logistic regression analysis. However, a possible confounder we were not able to adjust for was the use of multiple surgeons. Further, since this was a retrospective study, an *à priori* power analysis could not be performed. Moreover, the femoral tunnel is only one of the variables in a biomechanical system. There are other variables, such as the tibial tunnel and the properties of the graft, which affect the function of the reconstructed knee. The most important limitation is, of course, that the anatomic reconstruction principles were not known or adhered to at the time the study was conducted.

## Conclusion

The orientation of the femoral tunnel did not predict the long-term subjective outcome, functional outcome or the development of osteoarthritis in patients undergoing non-anatomic ACL reconstruction. The method for determining femoral rotation on lateral radiographs was found to be reliable.
